# Mapping the Complement Factor H-Related Protein 1 (CFHR1):C3b/C3d Interactions

**DOI:** 10.1371/journal.pone.0166200

**Published:** 2016-11-04

**Authors:** Jonathan P. Hannan, Jennifer Laskowski, Joshua M. Thurman, Gregory S. Hageman, V. Michael Holers

**Affiliations:** 1 Department of Medicine, University of Colorado School of Medicine, Aurora, Colorado, United States of America; 2 Sharon Eccles Center for Translational Medicine, John A. Moran Eye Center, Department of Ophthalmology and Visual Sciences, University of Utah, Salt Lake City, Utah, United States of America; University Medical Center Utrecht, NETHERLANDS

## Abstract

Complement factor H-related protein 1 (CFHR1) is a complement regulator which has been reported to regulate complement by blocking C5 convertase activity and interfering with C5b surface association. CFHR1 also competes with complement factor H (CFH) for binding to C3b, and may act as an antagonist of CFH-directed regulation on cell surfaces. We have employed site-directed mutagenesis in conjunction with ELISA-based and functional assays to isolate the binding interaction that CFHR1 undertakes with complement components C3b and C3d to a single shared interface. The C3b/C3d:CFHR1 interface is identical to that which occurs between the two C-terminal domains (SCR19-20) of CFH and C3b. Moreover, we have been able to corroborate that dimerization of CFHR1 is necessary for this molecule to bind effectively to C3b and C3d, or compete with CFH. Finally, we have established that CFHR1 competes with complement factor H-like protein 1 (CFHL-1) for binding to C3b. CFHL-1 is a *CFH* gene splice variant, which is almost identical to the N-terminal 7 domains of CFH (SCR1-7). CFHR1, therefore, not only competes with the C-terminus of CFH for binding to C3b, but also sterically blocks the interaction that the N-terminus of CFH undertakes with C3b, and which is required for CFH-regulation.

## Introduction

**Fig 1:**
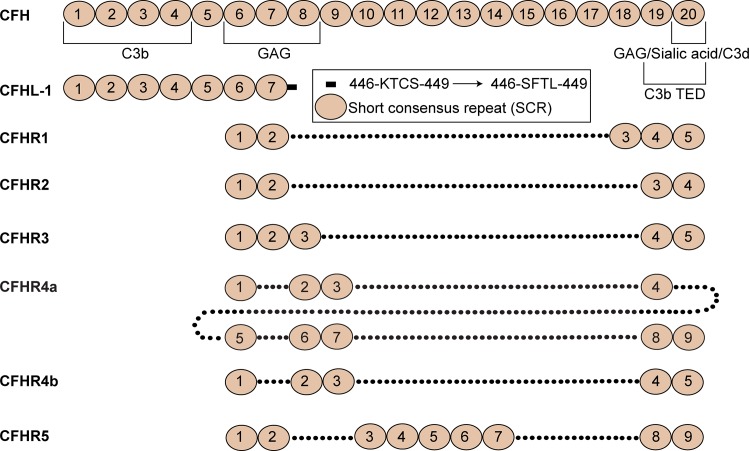
Domain composition of the *CFH* gene family. CFH comprises 20 SCR modules, and the *CFH* gene splice variant, CFHL-1, encompasses 7 SCR modules. CFHL-1 is invariant from the N-terminal seven SCRs of CFH apart from the C-terminal four residues (indicated). CFHR1-5 range from 4 to 9 SCRs. The SCR domains of each CFHR protein are aligned with the most homologous domains of CFH. C3b/C3d and GAG/sialic-binding regions are indicated. Two forms of CFHR4 are found in circulation, a long 9 SCR form of the protein, CFHR4a, and a shorter 5 SCR form, CFHR4b.

The proteins encoded by the *CFH* gene family include the complement regulator CFH as well as five CFH-related proteins, CFHR1, CFHR2, CFHR3, CFHR4 and CFHR5. All of these molecules are composed exclusively of compact repeating domains, each of about 60 residues, known as short consensus repeats (SCRs) or complement control protein modules (CCPs) [1, 2]. Human CFH is a 150 kDa plasma protein that is comprised of twenty SCR modules, which is found in circulation at concentrations ranging from ~0.8–3.8 μM (116–562 μg/ml) [3]. A more recent study has determined mean levels of CFH at ~1.6 μM (233 μg/ml) in young adults and at ~1.8 μM (269 mg/ml) in the elderly [4]. Alternative splicing of the *CFH* gene also results in the production of a truncated 42 kDa isoform of CFH known as complement factor H-like protein 1 (CFHL-1). With regards to the CFHR proteins, each of which are encoded by unique but closely linked genes (*CFHR3*, *CFHR1*, *CFHR4*, *CFHR2*, *CFHR5*), these molecules comprise between four and nine SCR domains (Fig 1).

CFH regulates the alternative pathway of complement in both the fluid phase and on self-surfaces: It competes with complement factor B (CFB) for binding to C3b and C3(H_2_O) thereby blocking the formation of the pro-convertase complexes, C3bB and C3(H_2_O)B. It also accelerates the decay of any existing C3bBb or C3(H_2_O)Bb. Finally, CFH is a cofactor for the complement factor I (CFI)-mediated cleavage of C3b to form iC3b, which cannot bind CFB. The C3b-regulatory and decay accelerating activity of CFH are located within the first four SCR domains (CFH SCR1-4), while the two C-terminal domains (CFH SCR19-20) bind C3b within a domain containing a reactive thioester (thioester-containing domain; TED) which facilitates the covalent attachment of C3b to surfaces via Gln1013 [5–12]. Two sites on CFH also localize this molecule to self-surface anionic markers such as glycosaminoglycans (GAGs) and sialic acid moieties: within CFH SCR6-8 (particularly SCR7) and SCR20 [9, 11, 13–16]. A site within SCR20 which overlaps closely with regions associated with GAG/sialic acid engagement has also been linked with binding to the complement C3 breakdown product, C3d (an opsonin) [6]. This putative C3d-binding site located on CFH SCR20 does not overlap with the SCR19-20 binding site which is involved in C3b TED engagement.

In contrast to CFH, the physiologic functions of CFHR1-5 are not clearly understood. For years these proteins have been considered as complement regulators in their own right; for example, CFHR1 has been reported to block C5 convertase activity and interfere with C5b surface deposition and terminal complement complex formation [17]. Various roles in C3 regulation have also been posited for CFHR2, CFHR3 and for CFHR5 [18–20]. By contrast, a separate study has shown that CFHR4 may in fact activate complement by acting as a platform for C3 convertase (C3bBb) assembly by binding C3b. The resulting CFHR4-complexed C3bBb convertase has been reported to be more resistant to CFH-driven decay than C3bBb on its own [21]. Recently, however, data have been presented which indicate that the human CFHR proteins may act as competitive antagonists of CFH [22, 23]. These studies also demonstrated that homo- and hetero-dimerization and oligomerization of CFHR1, CFHR2 and CFHR5 mediated by interactions between SCR1-2 of each of these molecules is important for their ability to compete with CFH for C3b-binding [22, 23]. It is also of note that none of the CFHR proteins exhibit any significant homology to the region of CFH (SCR1-4) that is required for effective complement regulation [5, 7, 8]. However, each of these molecules possesses varying degrees of sequence identity with regions of CFH that are involved in GAG/sialic acid-binding (SCR7, SCR20) and with C3b TED-binding (SCR19-20).

A growing body of literature has linked genetic variants in CFHR1 with human disease (reviewed in [24]). It is important, therefore, to fully understand the function of CFHR1 and its interactions with other complement proteins. The goal of the current work is to understand how one of the most prevalent CFHR proteins, CFHR1, which is reported to be found in plasma at a concentration of 0.7–2.5 μM (70–100 μg/ml) [17], engages the complement C3 proteolytic products, C3b and C3d. In its native state CFHR1 comprises 5 SCR modules made up of 330 amino acids with two N-glycosylation sites located at positions 126 and 194, respectively. The molecular weight of the glycosylated mature protein (residues 19–330) is 37–42 kDa. The three C-terminal SCRs of CFHR1 are required for C3b/C3d binding, however, it is unclear as to whether CFHR1 binds both C3b and C3d at the same location, or whether non-overlapping binding sites are involved in the engagement of each molecule [17, 23]. The three C-terminal SCR domains of CFHR1 (SCR3-5) are almost identical to the corresponding three C-terminal SCR modules of CFH (SCR18-20); the CFHR1*A isoform varies over only five residues within the last three SCRs. Three of these five residues are located within SCR3 (which corresponds to SCR18), while the remaining two are located within SCR5 (which corresponds to SCR20). The CFHR1*B isoform, on the other hand, varies only over two residues within the last three SCR domains. In this case SCR3 is indistinguishable to that of SCR18 of CFH, and the two variant residues are located within SCR5. These two residues are identical to their counterparts in the *A isoform.

In this study we have generated recombinant wild-type and mutant forms of human CFHR1 (corresponding to the CFHR1*A variant; Uniprot identifier: Q03591) and wild-type and mutant forms of C3d to evaluate the interface(s) that CFHR1 makes with the C3 fragments, C3b and C3d. We have also generated a mutant monomeric full-length form of CFHR1 to verify that dimerization/oligomerization of this protein is necessary for functionality. In total, it is hoped this study will assist us to determine whether CFHR1 may act as a CFH antagonist by competing with binding to the TED of C3b in an interaction mediated primarily through SCR4 of CFHR1, or additionally by competing with CFH binding to C3d in an interaction mediated through SCR5 of CFHR1, and concurrently, whether dimerization of CFHR1 is necessary for effective C3b/C3d binding to occur. In addition, the capacity of CFHR1 to compete with the *CFH* gene splice variant, CFHL-1 (which does not contain the conserved C3b TED binding site) is also assessed.

## Materials and Methods

### Expression of wild-type and mutant forms of complement factor H-related protein 1 (CFHR1), complement factor H (CFH) and complement factor H-like protein 1 (CFHL-1)

The codon-optimized (*Homo sapiens*) DNA sequences for human CFHR1 (comprising residues 19–330; Uniprot identifier: Q03591), human CFH (comprising residues 19–1231; Uniprot identifier: P08603-1) and human CFHL-1 (comprising residues 19–449; Uniprot identifier: P08603-2) which had been sub-cloned into the pDONR221 entry vector (Life Technologies Inc) were purchased from GeneArt. The engineered sequences additionally contained DNA encoding an Ig kappa chain leader sequence to facilitate secretion of the target protein, a Gly-Ala-Gly-Ala-Gly-Ala linker region, a hexa-histidine (His_6_-) fusion tag, a second linker region (Asp-Tyr-Asp-Ile-Pro-Thr-Thr) and a Tobacco Etch Virus nuclear inclusion A endopeptidase (TEV) cleavage site (Glu-Asn-Leu-Tyr-Gln-Gly), all of which are located 5′ prime to the synthetic genes. These sequences were then re-combined into a pcDNA3.2/V5-Dest expression vector using Gateway LR clonase II enzyme mix (Life Technologies Inc) according to the manufacturer’s instructions. The resulting expression constructs were then amplified and transiently transfected into Freestyle^TM^ 293f cells grown in suspension in an 8% CO_2_ humidified environment at 37°C. After 96 hours the cells were harvested by centrifugation, and the spent medium collected and passed through a 0.22 μm filter to remove cellular debris. The medium was then diluted into a 5X buffer containing 0.1 M sodium phosphate pH 7.8, 2.5 M NaCl, 0.1 M imidazole (to give a final working concentration of 20 mM sodium phosphate, pH 7.8, 0.5 M NaCl, 20 mM imidazole). Samples were then applied to a 5 ml Histrap HP column (GE Healthcare Inc) using an ÄKTAprime plus (GE Healthcare Inc) liquid chromatography system and bound His_6_-tagged CFHR1, CFH or CFHL-1 protein eluted using a linear imidazole gradient (20 mM to 0.5 M). The eluted proteins were then concentrated at room temperature using at a Vivaspin 20 device (Millipore Inc.), and applied to a HiPrep 16/60 Sephacryl size exclusion column (GE Healthcare Inc) (S200 for CFHR1 and CFHL-1, and S300 for CFH) which had been equilibrated with Dulbecco’s phosphate buffered saline without MgCl_2_ or CaCl_2_ (DPBS: 2.7 mM KCl, 1.5 mM KH_2_PO4, 138 mM NaCl, 8.1 mM Na_2_HPO_4_, pH 7.4) using an ÄKTADesign high pressure liquid chromatography system (GE Healthcare Inc).

To interrogate the interface through which CFHR1 interacts with the C3b TED we utilized the previously elucidated structure of the C-terminal two domains of wild-type CFH (SCR19-20) in complex with C3d to design two mutations which would likely perturb the corresponding binding interaction that CFHR1 undertakes with C3b or with C3d (the C3 fragment which approximates to the C3b TED) [9]. Plasmid DNA encoding CFHR1 Mut1 (N216A/D218A) and CFHR1 Mut2 (Q238A/Y241A) were generated using a QuikChange Lightning site-directed mutagenesis kit (Stratagene), according to the manufacturer’s instructions. After verification that the DNA contained the correct substitutions, the CFHR1 Mut1 and Mut2 plasmid DNA was amplified, transiently transfected into Freestyle^TM^ 293f cells and the proteins expressed and purified as described above. A third CFHR1 (CFHR1 Mut3) mutant protein was also generated which contains a triple Y52S/S54Y/Y57E mutation targeting a conserved dimerization interface that is found in CFHR1, CFHR2 and CFHR5.

### Expression of wild-type and mutant C3d recombinant proteins in *Escherichia coli*

Wild-type C3d, corresponding to residues 996–1306 of wild-type human C3 (prepro C3 numbering used throughout), was produced and purified as previously described [9, 25–27]. To investigate which residues on C3 contribute to the CFHR1:C3b TED binding interaction we also used previously generated [9, 25], or newly engineered mutant forms of C3d which were produced using a Quikchange Lightning mutagenesis kit. New mutations were directed using the available crystal structure of the wild-type CFH SCR19-20:C3d complex [9]. A total of twelve mutant forms of C3d were utilized for this study (Table 1). All mutant forms of C3d were expressed and purified as previously described above for the wild-type protein.

**Table 1:** Residues on CFHR1 and C3d selected for mutagenesis screening.

Mutations	Mutagenesis Primers
**CFHR1 Mutations targeting C3b TED Binding Site and Dimer Interface**	
CFHR1 Mut1: N216A/D218A	5′ CC ATC GAC GCT GGC GCC ATC ACC AGC TTC 3′
	5′ GAA GCT GGT GAT GGC GCC AGC GTC GAT GG 3′
CFHR1 Mut2: Q238A/Y241A	5′ GAA TAC CAG TGC GCG AAT CTG GCC CAG CTG GAA GGC 3′
	5′ GCC TTC CAG CTG GGC CAG ATT CGC GCA CTG GTA TTC 3′
CFHR1 Mut3:Y52S/S54Y/Y57E	5ʹ GAG GTG TTC TCC TAC TAC TGC GAG GAA AAC TTC GTG TCC 3ʹ
	5ʹ GGA CAC GAA GTT TTC CTC GCA GTA GTA GGA GAA CAC CTC 3ʹ
**C3d Mutations targeting C3b TED Binding Site**	
C3d: K1105A	5′ GC GGG GCT GTT GCA TGG CTG ATC 3′
	5′ GAT CAG CCA TGC AAC AGC CCC GC 3′
C3d: E1110A †,*	5′ GGC TGA TCC TGG CGA AGC AGA AGC CC 3′
	5′ GGG CTT CTG CTT CGC CAG GAT CAG CC 3′
C3d: E1110A/D1115A *	5′ GG CTG ATC CTG GCG AAG CAG AAG CCC GCC GGG GTC TTC CAG 3′
	5′ CTG GAA GAC CCC GGC GGG CTT CTG CTT CGC CAG GAT CAG CC 3′
C3d: P1114L	5′ GAG AAG CAG AAG CTC GAC GGG GTC TTC 3′
	5′ GAA GAC CCC GTC GAG CTT CTG CTT CTC 3′
C3d: D1115A †,*	5′ GCA GAA GCC CGC CGG GGT CTT CCA G 3′
	5′ CTG GAA GAC CCC GGC GGG CTT CTG C 3′
C3d: N1163A †	5′ GCG AGG AGC AGG TCG CCA GCC TGC CAG GCA GC 3′
	5′ GCT GCC TGG CAG GCT GGC GAC CTG CTC CTC GC 3′
C3d: K1171Q	5′ GGC AGC ATC ACT CAA GCA GGA GAC TTC 3′
	5′ GAA GTC TCC TGC TTG AGT GAT GCT GCC 3′
**C3d Mutations targeting C3d Binding Site**	
C3d: D1029A †	5′ GTG CAT TAC CTG GCA GAA ACG GAG CAG 3′
	5′ CTG CTC CGT TTC TGC CAG GTA ATG CAC 3′
C3d: E1153A †,*	5′ CTC GCT GCA GGC GGC TAA AGA TAT TTG 3′
	5′ CAA ATA TCT TTA GCC GCC TGC AGC GAG 3′
C3d: D1156A †	5′ CAG GAG GCT AAA GCT ATT TGC GAG GAG 3′
	5′ CTC CTC GCA AAT AGC TTT AGC CTC CTG 3′
C3d: D1156A/I1157A †	5′ CAG GAG GCT AAA GCC GCT TGC GAG GAG CAG 3′
	5′ CTG CTC CTC GCA AGC GGC TTT AGC CTC CTG 3′
C3d: D1285A	5′ CTG CAG CTA AAA GCC TTT GAC TTT GTG 3′
	5′ CAC AAA GTC AAA GGC TTT TAG CTG CAG 3′

Shown are the primers utilized to generate three multiple-site mutant forms of CFHR1 and ten single-site and two multiple-site mutant forms of recombinant C3d which were employed to target both the C3b TED/C3d interaction and an additional proposed CFH SCR20-interaction site located within C3d. Mutants indicated with a ‘†’ have previously been used in [25], while those marked with a ‘*’ have previously been used in [9].

### Wild-type and mutant CFHR1-C3d/C3b binding assay

Plates were coated overnight at 4°C with 14.1 pmols of recombinant wild-type C3d expressed and purified as described above, or with 5.5 pmols of commercially available serum-derived C3b (CompTech Inc) in 50 mM sodium bicarbonate buffer, pH 8.8. After coating, the plates were blocked using 1% BSA in DPBS for at least one hour at room temperature. The plates were then washed three times using DPBS-Tween 20 (0.005%), and incubated with wild-type or mutant forms (Mut1: N216A/D218A, Mut2: Y238A/Y241A and Mut3: Y52S/S54Y/Y57E) of CFHR1 at concentrations ranging from 1.04–0.008 μM in DPBS for one hour at room temperature. After further washing, plates were incubated with a commercially available horseradish peroxidase (HRP)-conjugated anti-His_6_ polyclonal antibody (Abcam: ab1187) at a 1/1000th dilution in DPBS for one hour. CFHR1-binding to the plate-bound C3d or C3b was subsequently detected utilizing ABTS (2,2'-azino-bis(3-ethylbenzthiazoline-6-sulphonic acid). In each case OD values obtained at 405 nm were normalized to the reading obtained for wild-type CFHR1 at a concentration of 1.04 μM.

### Mutant C3d-CFHR1 binding assay

ELISA-based experiments were carried out in which wells were coated side by side with 14.1 pmols of recombinant wild-type or mutant forms of C3d overnight at 4°C. CFHR1-binding was then assessed using HRP-conjugated anti-His_6_ polyclonal antibody and ABTS as described above. In each case, binding of mutant C3d proteins was normalized to OD values obtained at 405 nm for CFHR1 (at a concentration of 1.04 μM) binding to wild-type C3d.

### C3c binding assay

Commercially available C3c (CompTech Inc) was used to coat plates overnight at 4°C, at concentrations of 18 pmols, 36 pmols and 72 pmols in 50 mM sodium bicarbonate buffer, pH 8.8. After coating, the plates were washed with DPBS-Tween 20 (0.005%) then blocked with 1% BSA, as described above. After further washing plates were incubated with wild-type CFHR1 at concentrations ranging from 1.04–0.008 μM in DPBS for one hour at room temperature. Plates were then incubated with HRP anti-His6 polyclonal antibody and CFHR1-binding assessed using ABTS.

### CFH competition assay

Plates were coated overnight at 4°C with 5.5 pmols of wild-type C3b (CompTech Inc) in 50 mM sodium bicarbonate buffer, pH 8.8. After coating, plates were blocked with 1% BSA in DPBS, pH 7.4 for one hour at room temperature. Plates were then washed with PBS-Tween 20 (0.005%). A solution comprising 0.14 μM of recombinant CFH was added to half of the C3b-coated wells to act as a positive control. To the other half of the C3b-coated wells 0.14 μM of recombinant CFH additionally containing wild-type or mutant forms (Mut1, Mut2, or Mut3) of recombinant CFHR1 at concentrations ranging from 2.9–0.09 μM in DPBS were added. After a one hour incubation period the plates were washed and then incubated with the anti-CFH monoclonal antibody, OX24 (Genway Biotech Inc), at a dilution of 1/1000 dilution in DPBS (working concentration 1 μg/ml) for one hour at room temperature, followed by further washing. Plates were then incubated for one hour with HRP-conjugated Goat-anti-mouse IgG (heavy and light chain) (Jackson Immunoresearch Inc.) at a 1/2000 dilution in DPBS. CFH binding was then detected using ABTS as described above for the C3d/C3b-binding assay. OD values at 405 nm were expressed as a percentage of CFH binding whereby readings where CFH was incubated in the absence of wild-type or mutant CFHR1 proteins was considered 100%.

### CFHL-1 competition assay

Plates were coated overnight at 4°C with 5.5 pmols of wild-type C3b (CompTech Inc) in 50 mM sodium bicarbonate buffer, pH 8.8 as described above. After coating, plates were blocked with 1% Fatty-Acid free-BSA in DPBS, pH 7.4 for one hour at room temperature. Plates were then washed with PBS-Tween 20 (0.005%). A solution comprising 0.19 μM of recombinant CFHL-1 was added to half of the C3b-coated wells to act as a positive control. To the other half of the C3b-coated wells 0.19 μM of recombinant CFHL-1 additionally containing CFHR1 at concentrations ranging from 7.6–0.24 μM in DPBS were added. CFHL-1 binding in the absence and presence of CFHR1 was detected as described above for the CFH competition assay. Background binding of CFHL-1 to Fatty-Acid free-BSA was subtracted from all values prior to normalization of data.

### Heparin pull-down

Wild-type C3d (1.1 nmols) and wild-type CFHR1 (0.8 nmols) were re-suspended in 60 μl of DPBS and then incubated with an equal volume of a 50% slurry of Heparin Sepharose 6 Fast Flow media (GE Healthcare Inc) in DPBS for 30 minutes at room temperature. Separate conditions were also set up in which C3d (1.1 nmols) or CFHR1 (0.8 nmols) proteins alone were incubated with the Heparin Sepharose media. After the incubation period, the Heparin Sepharose beads were sedimented by centrifugation, and the supernatant aspirated. The beads were then washed three times with 200 μl of DPBS. After washing, the Heparin Sepharose beads were incubated with 20 μl of an elution buffer comprising DPBS supplemented with 1M NaCl at room temperature for 15 minutes. The beads were again sedimented, and the supernatants collected and analyzed by SDS PAGE. Gels were stained using a Colloidal Blue Staining Kit (Life Technologies Inc).

### Hemolysis Assay

An alternative pathway hemolysis assay was carried out using a modification of a previously described protocol [23]. Briefly 1 X 10^6^ guinea pig erythrocytes were suspended in 10% normal human serum (NHS) (CompTech Inc) in a hemolysis buffer comprising 20 mM Hepes, pH 7.3, 141 mM NaCl, 0.1% gelatin, and 5 mM Mg-EGTA. Additional conditions were also set up in which erythrocyte/NHS mixtures were supplemented with wild-type CFH (140 nM) (CompTech), or with 140 nM CFH plus one of CFHR1, CHFR1 Mut1, CFHR1 Mut2 or CFHR1 Mut3 each at a concentration of 2 μM in DPBS. Each of these experimental conditions were carried out on ice. The total volume for each reaction mix was 50 μl (reactions not supplemented with CFH/CFHR1 proteins additionally contained DPBS). Erythrocyte hemolysis was carried out by incubating reaction mixtures at 37°C for 30 minutes, after which each reaction was terminated by the addition of 150 μl of a chilled quenching buffer (20 mM Hepes, pH 7.3, 145 mM NaCl, 5 mM EDTA). Reactions were then clarified by centrifugation, and the supernatant collected for measurement. Readings were taken at an absorbance of 412 nm. Four replicates for each reaction were carried out, and data are presented as hemolysis relative to 100% lysis in water.

## Results and Discussion

**Fig 2:**
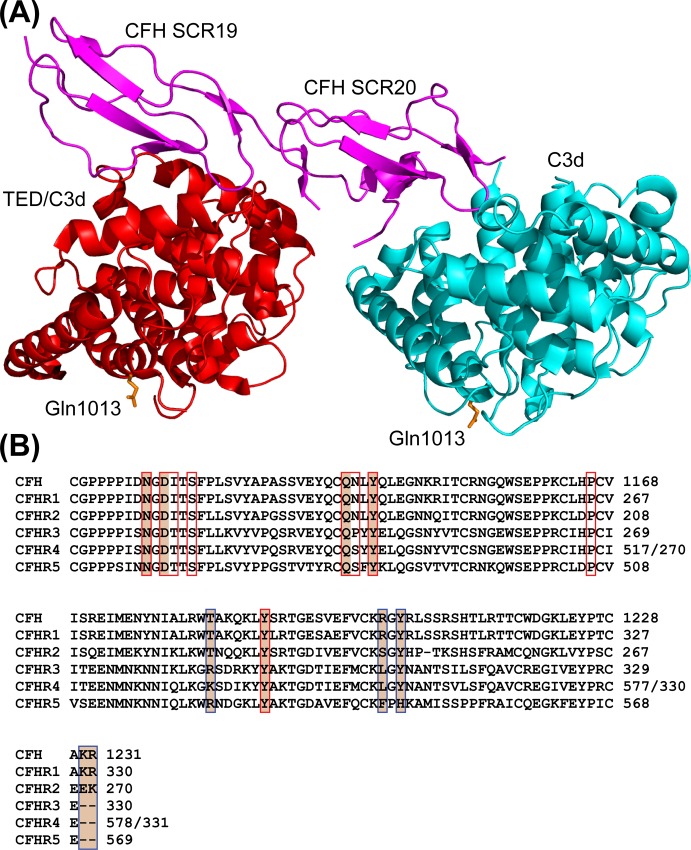
C3b TED/C3d-binding sites on CFH SCR19-20 and their conservation within the CFHR proteins. **(A)** Ribbon representations of CFH SCR19-20 in complex with C3d (corresponding to the C3b TED) and a separate C3d interaction. Shown is a superposition of the available structures of wild-type CFH SCR19-20 in complex with C3d and a D1119G/Q1139A double mutant form of CFH SCR19-20 in complex with C3d (PDB ID codes: 3OXU and 2XQW, respectively) [6, 9]. Wild-type CFH SCR19-20 is represented in magenta, and the C3d molecule most likely to approximate to that of the C3b TED is shown in red (both taken from 3OXU). A second proposed interaction with C3d is also shown, with the C3d molecule in this case being colored cyan (taken from 2XQW). For the purposes of this representation, the structure of D1119G/Q1139A CFH SCR19-20 is not shown, nor is a similar but not identical second C3d interaction observed in the 3OXU structure. Gln1013, which is the point of covalent attachment of C3 moieties to activating surfaces, is indicated in orange for both C3d molecules. All structural representations have been generated using the PyMOL Molecular Graphics System, Version 1.7.0.1 (Schrödinger, LLC). **(B)** Sequence alignment of the two C-terminal SCR domains of CFH with the corresponding two C-terminal SCR modules of each of the CFHR proteins (CFHR1-5). Indicated within red boxes are those residues of CFH which are involved in interface-formation with a C3d molecule which can be approximated to the C3b TED. Red boxes which are shaded in tan indicate those residues of CFH SCR19-20 which contribute side-chains to intermolecular hydrogen bonds [9]. Indicated in blue boxes are those residues which contribute to the interface of an additional proposed physiologically important interaction that CFH SCR20 undertakes with a separate C3d molecule. Blue boxes which are shaded in tan indicate those residues of CFH SCR20 which contribute side-chains to intermolecular hydrogen bonds and/or salt-bridges [6].

The mechanism by which CFHR1 engages the complement C3 components, C3b and C3d has not previously been established. The C-terminal three SCR domains (SCR3-5) of CFHR1, however, are highly homologous to the corresponding C-terminal SCRs of CFH (SCR18-20). The primary interaction site for C3b on CFH, which is required for CFH-mediated complement regulation is located within SCR1-4 [28]. However a second binding-site on CFH which interacts directly with the C3b TED is housed within SCR19-20 [6, 9, 15]. A separate putative C3d interaction site has also been identified within SCR20 of CFH [6]. With regards to the C-terminal CFH:C3b TED binding site, two independently derived three-dimensional structures of wild-type and mutant forms of CFH SCR19-20 in complex with C3d (which approximates to the TED) have been reported, which delineate essentially the same interface, and which is widely accepted as being reflective of the physiologic CFH:C3b TED interaction. The TED-binding site on CFH mainly involves contributions from residues within SCR19 (but with additional interactions also being provided by the linker region connecting SCR19 and SCR20, and also by SCR20) (Fig 2 and S1 Fig) [6, 9]. This interaction is also observed in an elucidated structure of a sialyl lactose trisaccharide (Neu5Acα2-3Galβ1-4Glc):CFH SCR19-20:C3d ternary complex [15]. All three of these complex datasets also exhibit a broadly similar interaction in which CFH SCR20 engages an additional C3d molecule at a binding site on CFH that is disparate from that of the C3b TED-binding site (Fig 2 and S1 Fig). When mapped onto the surface of the crystal-derived structure of unbound C3b, this second CFH binding surface is partially occluded by a neighboring macroglobulin domain (MG1) meaning that this site would only be accessible for CFH-engagement in the context of C3d, but not the larger C3b molecule [29]. It should be noted, however, that there is accumulating evidence that under solution conditions C3b is a flexible entity in which the TED can adopt a range of conformations with relation to the rest of C3b (reviewed in [30]). Kajander and colleagues have posited that this second C3d-binding site is physiologically important, with the ability of the C-terminus of CFH to simultaneously engage C3b and C3d resulting in a similar phenotype to that which occurs when CFH forms a ternary complex with C3b and anionic markers such as GAGs and sialic acid moieties. That is, effective CFH-mediated complement regulation occurs on surfaces and tissues decorated with both of these entities. Indeed, some support for the dual recognition of C3b/C3d by CFH has been derived from data indicating that mutant forms of CFH SCR19-20, and of C3dg or C3d, in which residues have been substituted within the CFH SCR19-20:C3b TED or the CFH SCR20:C3d interaction sites result in substantially altered binding affinities compared to those of the wild-type proteins. Additionally, mutant forms of CFH SCR19-20 again separately targeting these interaction sites demonstrate altered abilities in their capacities to act as antagonists of CFH in functional assays (S1–S4 Tables) [6, 9, 31, 32].

Those residues on CFH which contribute side-chain interactions to the CFH SCR19-20:C3b TED interface are highly conserved in CFHR1 suggesting that this molecule may bind to the C3b TED at an identical binding site to that of CFH SCR19-20 (Fig 2). This would be supportive of data which indicate that under certain physiologic conditions some of the CFHR proteins (CFHR1, CFHR2 and CFHR5) may act as competitive antagonists of CFH [22, 23]. In addition, CFHR1 alone of all the CFHR proteins has excellent sequence identity with the additional putative C3d-binding site located within CFH SCR20 (Fig 2).

**Fig 3:**
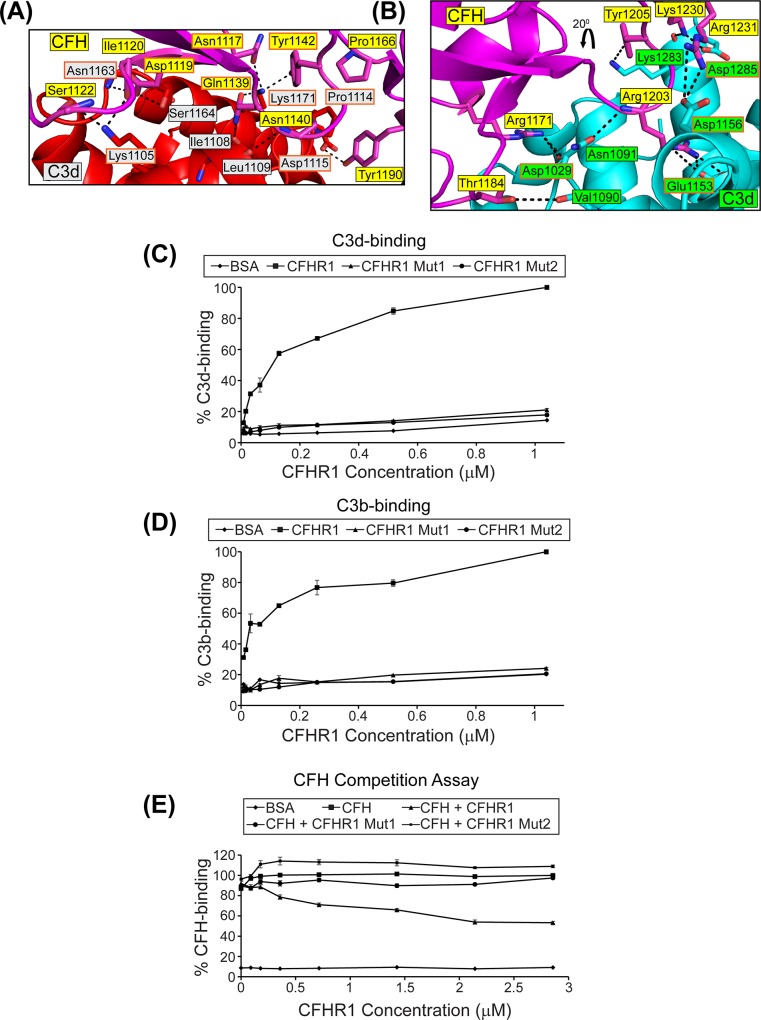
Mutagenesis data targeting the C3b TED and C3d interfaces. **(A)** Close-up of the CFH SCR19-20:C3d/C3b TED interface generated from the wild-type CFH SCR19-20:C3d crystal structure [9]. The complex is in the same orientation as that represented in Fig 2. CFH SCR19-20 and C3d are colored magenta and red, respectively. Intermolecular hydrogen bonds are indicated by black dashed lines. This structure was used to direct alanine substitutions in CFHR1 targeting residues Asn216 and Asp218, corresponding to Asn1117 and Asp1119 in CFH (CFHR1 Mut1: N216A/D218A) and also residues Gln238 and Tyr241, corresponding to Gln1139 and Tyr1142 in CFH (CFHR1 Mut2: Q238A/Y241A). Labels for CFH residues are shown in yellow boxes, and labels for C3d residues are shown in grey boxes. For clarity, residues within CFHR1 and C3d which were selected for mutagenesis studies are delineated by orange boxes. **(B)** Close-up of a second possible CFH SCR20:C3d interaction site generated from the CFH SCR19-20 D1119G/Q1139A:C3d crystal structure [6]. In this case, the additional C3d moiety is colored cyan, with labels for residues from this molecule highlighted in green boxes. Again, residues which were targeted for mutagenesis are highlighted within orange boxes. The complex has been rotated by 20 degrees in the y-axis to that represented in Fig 2. Intermolecular hydrogen bonds and salt-bridges are indicated by black dashed lines. **(C)** and **(D)** The abilities of wild-type CFHR1 and mutant CFHR proteins to bind to plate-bound recombinant C3d and serum-derived C3b, respectively. Obtained OD_405_ readings were normalized to values obtained for wild-type CFHR1 binding at a concentration of 1.04 μM to C3d or C3b. **(E)** The ability of wild-type and mutant forms of CFHR1 to compete with CFH-binding to plate-bound serum-derived C3b are shown. CFH-binding is inhibited in the presence of wild-type CFHR1 in a dose dependent manner. However, in the presence of the CFHR1 Mut1 and CFHR1 Mut2 proteins, no inhibition of CFH-binding to C3b can be observed. OD values at 405 nm were expressed as a percentage of CFH binding where CFH incubated in the absence of wild-type or mutant CFHR1 proteins was considered 100%.

Our recombinant wild-type CFHR1 protein was able to bind plate-immobilized serum-derived C3b and recombinant C3d in a dose dependent manner. It was also able to compete with wild-type CFH for binding to C3b consistent with previously reported data [22, 23]. (Fig 3). To interrogate the interaction that occurs between CFHR1 and complement components C3b and C3d we used the available structure of the wild-type CFH SCR19-20:C3b TED complex to generate two double mutant forms of CFHR1. CFHR1 Mut1 and CFHR1 Mut2 (Mut1: N216A/D218A and Mut2: Q238A/Y241A) were designed to disrupt the interface between CFHR1 and the C3b TED. The corresponding wild-type residues on CFH, Asn1117 and Asp1119, and Gln1139 and Tyr1142, respectively, are involved in critical intermolecular interactions with C3d (Fig 3) [9]. The side-chains of all of the CFHR1 residues targeted by site-directed mutagenesis exhibit high solvent accessibility and are therefore unlikely after substitution, to result in significant structural perturbations to the overall protein fold [33]. However, neither the CFHR1 Mut1 nor the CFHR1 Mut2 proteins bound to plate-immobilized C3b or C3d effectively. Indeed, the levels of binding that was observed for these two CFHR1 mutants were similar to those recorded for wild-type CFHR1-binding to BSA (Fig 3). Mutations targeting the C3b TED interface in the shorter isoform of CFHR4 (CFHR4B) also exhibit reduced (a D221G substitution, targeting the residue corresponding to Asp1119 on CFH) or negligible binding (a D221G/K290A double substitution targeting residues corresponding to Asp1119 and Lys1188 on CFH) to C3b [21]. The D221G/K290A mutant was also ineffective at promoting alternative pathway-mediated complement activation [21].

**Fig 4:**
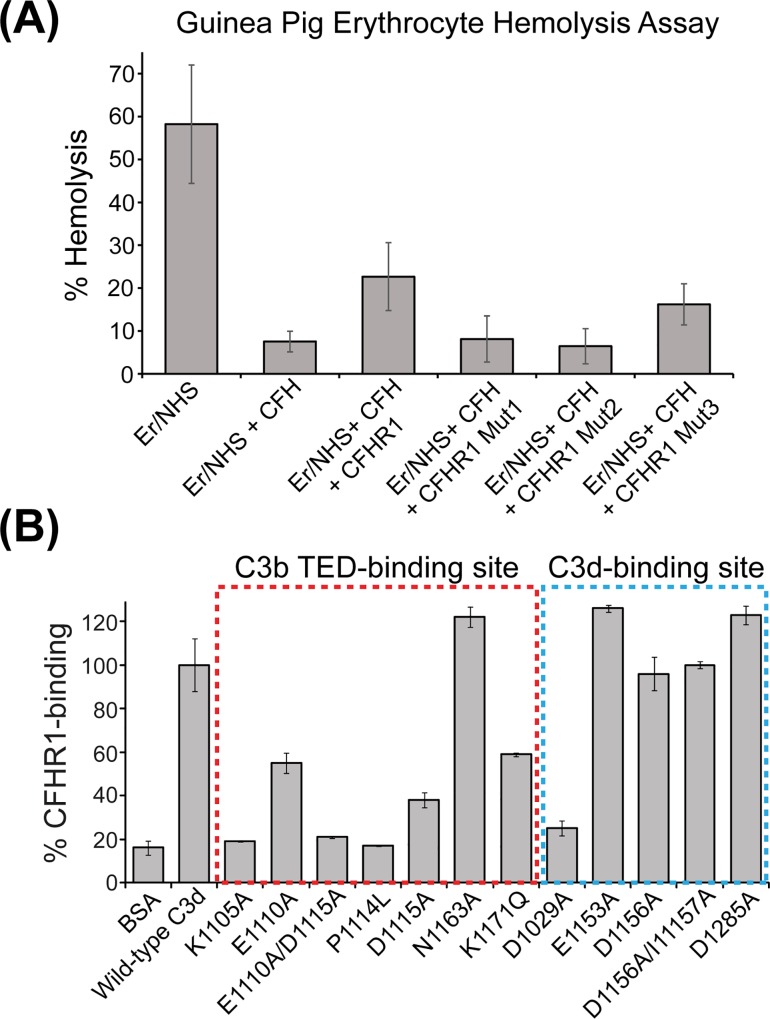
**(A)** Capacity of wild-type and mutant forms of CFHR1 to inhibit CFH-mediated protection of guinea pig erythrocytes from complement-driven cell lysis. CFH-binding to C3b deposited on guinea pig erythrocytes, with concomitant protection from hemolysis, is reduced by the presence of wild-type CFHR1. However, CFH-mediated protection of erythrocytes is maintained in the presence of CFHR1 Mut1 and Mut2 proteins, and to a lesser extent in the presence of CFHR Mut3. **(B)** Summary of wild-type CFHR1 binding to mutant forms of C3d targeting both the C3b TED-binding site (indicated within a red box) and the second possible C3d-binding site (indicated within a blue box). Given are percentage values of CFHR1-binding to plate-bound mutant C3d relative to that with wild-type C3d at a concentration of 1.04 μM.

These ELISA-derived data are consistent with an alternative pathway hemolysis assay in which unopsonized guinea pig erythrocytes were incubated in the presence of 10% NHS (Fig 4). The addition of 140 nM of recombinant CFH substantially reduced the levels of hemolysis from ~58% to ~8%. However, upon addition of 140 nM of recombinant CFH also supplemented with 2 μM wild-type CFHR1, the level of hemolysis was only reduced to ~23%, indicating that wild-type CFHR1 was able to compete with CFH for binding to C3b which had been deposited on the surface of the guinea pig erythrocytes. Finally, when CFHR1 Mut1 or CFHR1 Mut2 were separately added to the supplemented CFH, the levels of hemolysis remained at 8% and 6%, respectively, similar values to that obtained for CFH alone, indicating that these mutant proteins were ineffective at competing with CFH for binding to C3b.

To precisely delineate the nature, and identify any differences between the CFHR1:C3b TED and CFHR1:C3d interactions we utilized mutant forms of recombinant C3d encompassing both the TED/C3d and the additional putative C3d-binding sites. A total of twelve novel or previously generated forms of C3d were expressed (Table 1) [9, 25]. Seven of these mutations targeted the primary C3b TED/C3d-binding interaction: K1105A, E1110A, E1110A/D1115A, P1114L, D1115A, N1163A and K1171Q. Five additional mutations targeted the second C3d-specific binding interaction: D1029A, E1153A, D1156A, D1156A/I1157A and D1285A. Of the seven mutations directed against the C3b TED/C3d-binding site, six mutations (K1105A, E1110A, E1110A/D1115A, P1114L, D1115A, and K1171Q) demonstrated significantly reduced binding for wild-type CFHR1 (Fig 4). In particular, the K1105A, E1110A/D1115A and P1114L C3d mutants exhibited binding levels comparable to background BSA-binding. Only the N1163A C3d mutant exhibited wild-type levels of CFHR1 binding. Interestingly, in a previous study, the N1163A mutant also exhibited wild-type levels of binding to CFH SCR19-20, likely because in the CFH SCR19-20:C3d complex structure, N1163A appears to contribute significantly to the interface only by means of main-chain contributions [9]. By contrast, only a single mutation, D1029A, targeting the C3d-specific interaction resulted in a substantial decrease in CFHR1-binding. The four other mutants, E1153A, D1156A, D1156A/I1157A and D1285A, all exhibited wild-type levels (or greater) of binding (Fig 4).

In summary, the CFHR1 Mut1 and Mut2 proteins were able to completely abrogate CFHR1-binding to both C3b and C3d, and effectively abolished the ability of CFHR1 to compete with CFH-binding to C3b. Also, all but one of the C3d mutations targeting the shared C3b TED/C3d interaction site resulted in decreased or negligible binding to CFHR1. By contrast, only a single C3d mutation targeting the C3d-specific binding site resulted in reduced CFHR1-binding. While we cannot unequivocally state that CFHR1 binds to both the C3b TED and to C3d solely at a single binding site which is identical to that described by the CFH SCR19-20:C3b TED interaction, this certainly looks like the most likely explanation for our data. The sole deleterious C3d mutation targeting the possible CFHR1 SCR5:C3d interaction site may simply be the result of a structural perturbation in the expressed protein.

There is support for the hypothesis that CFHR1 binds to both C3b and to C3d at a single binding site in an interaction mediated primarily by SCR4 (with additional contributions from SCR5), and that this molecule does not additionally engage C3d via a discrete region on SCR5. A recent study by Buhlmann and colleagues has shown that CFHR3 is able to disrupt the interaction that occurs between serum-derived C3d and recombinant complement receptor type 2 (CR2; CD21) SCR1-4 and can also abrogate C3d-mediated signaling in Raji cells and in peripheral B cells by blocking binding of C3d-opsonized antigen to the B cell coreceptor (CR2/CD19/CD81) complex. Importantly, CFHR1 was unable to significantly disrupt the interaction between C3d and CR2 SCR1-4, and was also unable to inhibit signaling in either Raji cells or in peripheral B cells [34]. The likely physiological CR2-binding site on C3d has been isolated by both site-directed mutagenesis and also by the elucidation of a three dimensional CR2 SCR1-2:C3d complex to a site that overlaps directly with that delineated by the CFH SCR20:C3d interaction identified in the various crystal-derived structures (S1 Fig) ([25, 35–37] and reviewed in [38]). As such, if CFHR1 SCR5 was able to bind to C3d with high affinity we would anticipate that it would be able to directly block the interaction between C3d and CR2 and, like CFHR3, disrupt signal transduction driven by the B cell coreceptor complex in cell-signaling assays (S1 Fig); in this particular study CFHR1 and CFHR3 were found to bind to C3d immobilized on a Ni-NTA biosensor chip with almost identical affinities, as measured by biolayer interferometry (*K*_D_ for CFHR1 = 1.2 μM, *K*_D_ for CFHR3 = 1.1 μM) [34].

**Fig 5:**
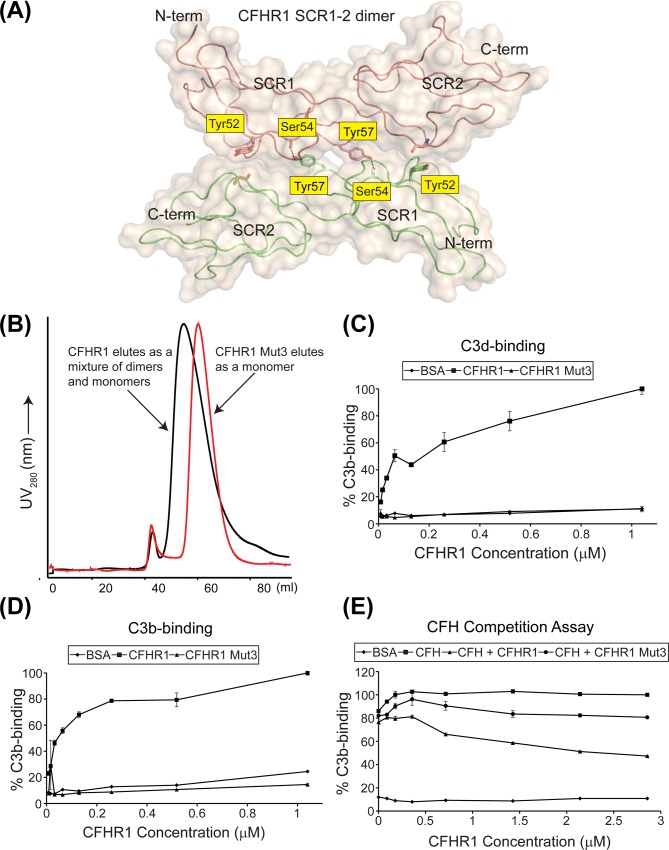
Mutagenesis data targeting the CFHR1:CFHR1 dimer interface. **(A)** Structure of the CFHR1 SCR1-2:CFHR1 SCR1-2 dimer (PDB ID code: 3ZD2) [23]. Indicated are residues (Tyr52, Ser54 and Tyr57) which play essential roles in dimer formation. **(B)** Size exclusion profiles of CFHR1 (black) and CFHR1 Mut3 (red). CFHR1 elutes from a HiPrep Sephacryl S200 16/60 HR column with a profile consistent with variably glycosylated dimers and monomers, while CFHR1 Mut3 elutes with a profile consistent with that of a glycosylated monomer. **(C)** and **(D)** The ability of wild-type CFHR1 and CFHR Mut3 proteins to bind to plate-bound recombinant C3d and serum-derived C3b, respectively. **(E)** The ability of wild-type CFHR1 and CFHR1 Mut3 to compete with CFH for binding to plate-bound serum-derived C3b. In this case, CFHR1 Mut3 protein is less effective at disrupting the CFH:C3b interaction than wild-type CFHR1.

Native CFHR1, CFHR2 and CFHR5 circulate as homo- and hetero-dimeric and oligomeric complexes [22, 23]. Dimerization of these molecules is facilitated by means of a conserved motif present within SCR1-2 of each of these molecules. In particular, Tyr52, Ser54 and Tyr57 within SCR1 are critical in stabilizing dimer formation (Fig 5). It is likely that the mode of dimerization of CFHR1 is distinct from that reported for CFH: Previous studies have indicated that a low percentage (5–14%) of CFH self-associates under physiological ionic strength conditions in interactions which are mediated by SCR6-8 and SCR16-20 (although other SCR modules may also be involved) [39–41]. CFH oligomerization may also be influenced by a number of additional factors, including the presence of divalent ions such as zinc or copper, binding to C3d, and by interactions with heparin moieties (reviewed in [42]). However, while CFH SCR6-7 share approximately 41% sequence identity with CFHR1 SCR1-2, CFH lacks the conserved dimerization motif present in CFHR1, CFHR2 and CFHR5. The critical importance of Tyr52, Ser54 and Tyr57 in CFHR dimer formation (and on protein functionality) has been highlighted by a recombinant form of CFHR5 in which these residues were substituted to the corresponding residues on CFH SCR6 (Y52S/S54Y/Y57E). Mutation of these residues resulted in a monomeric form of CFHR5 that was unable to bind effectively to activated mouse C3 within the glomerular basement membrane in a CFH-deficient mouse model [23]. Monomeric forms of CFHR1 and CFHR2 comprising SCR3-5 and SCR3-4, respectively, and which therefore lack those modules which are involved in dimer formation are also less able to compete with CFH for binding to C3b, or to deregulate CFH function in an erythrocyte hemolysis assay [21].

To confirm the importance of dimerization for CFHR1 function we generated a CFHR1 Y52S/S54Y/Y57E triple substitution (CFHR1 Mut3) similar to that described above for CFHR5. Wild-type CFHR1 eluted from a preparative size-exclusion column with a profile consistent with a variably glycosylated mixture of dimers and monomers, similar to that which has been described for native CFHR1 in serum. By contrast the CFHR1 Mut3 protein eluted with a profile consistent with that of the protein existing primarily as a monomer (Fig 5). The resulting purified Mut3 protein exhibited negligible or substantially reduced ability to bind to C3d or to C3b, respectively, in a plate-based assay, presumably as a result of reduced avidity. The CFHR1 Mut3 protein was also less able to act as an antagonist of CFH in a competition-based assay or in our functional hemolysis assay (Fig 4 and Fig 5). In the case of the hemolysis assay, the addition of 140 nM of recombinant CFH supplemented with 2 μM CFHR1 Mut3 to 10% NHS resulted in ~12% hemolysis, indicating that while likely less effective at inhibiting CFH than wild-type CFHR1 (23%) (substantial experimental errors were seen for these data), it retained greater functionality than CFHR1 Mut1 or CFHR1 Mut2. These data are consistent for those reported for other monomeric CFHR1, CFHR2 and CFHR5 constructs, which also exhibit a reduced capacity to deregulate CFH [21]. Indeed, it appears that the greater the oligomeric size of CFHR complexes the more effective their abilities to act as CFH antagonists, and the greater their affinities for C3 entities: A naturally occurring mutant form of CFHR1 has been identified in which SCR1-4 are duplicated in tandem forming a SCR123412345 motif. This molecule forms abnormally large multimeric complexes which exhibit increased avidity for C3b. This mutant CFHR1 protein exhibits much slower dissociation from C3b than wild-type CFHR1, and the gene encoding this duplication associates with the development of pathology in the kidney characterized by glomerular inflammation and enhanced C3 deposition (C3 glomerulopathy; C3G) [22]. An internal duplication of the gene encoding CFHR5 which also associates with C3G has additionally been identified [43]; in this case SCR1-2 are duplicated at the N-terminus of the encoded protein resulting in an eleven SCR protein (SCR12123456789) which contains two dimerization sites. Like the mutant form of CFHR1 described above, this CFHR5 entity also exhibits a propensity to form abnormally large oligomeric complexes, and is more potent than wild-type CFHR5 at deregulating CFH function [23].

**Fig 6:**
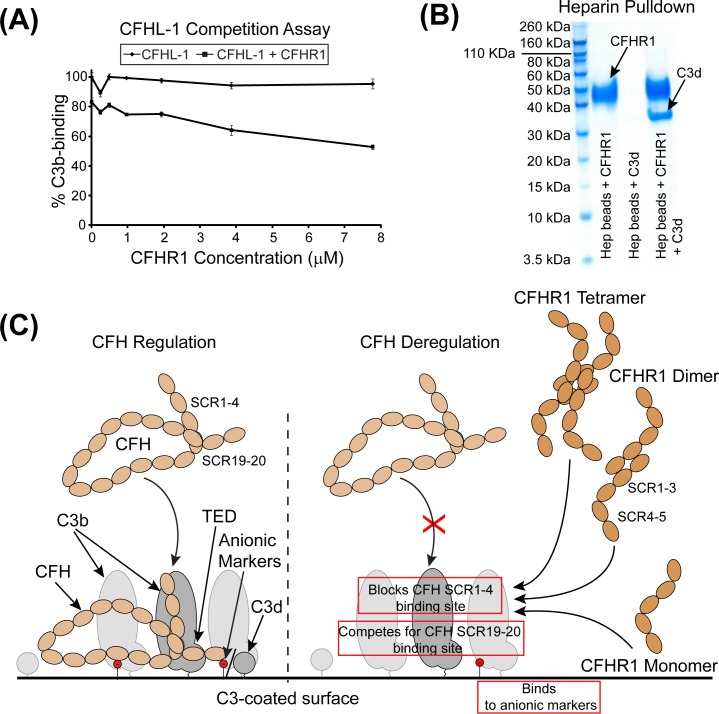
CFHR1 blocks other CFH-interaction sites on C3b. **(A)** The capacity of CFHR1 to compete with CFHL-1-binding to plate-bound C3b is shown. OD values at 405 nm were expressed as a percentage of CFHL-1 binding where CFHL-1 incubated in the absence of CFHR1 was calculated as 100%. **(B)** Heparin sepharose is able to pull-down CFHR1 (Left), but is unable to pull-down C3d on its own (Centre). However, when both CFHR1 and C3d are present, heparin-conjugated beads are able to pull-down both CFHR1 and C3d (Right). **(C)** Proposed mechanism of CFH regulation of C3b on a non-activating surface and CFHR1-driven deregulation of CFH on an activating surface. (Left) CFH engages C3b at two sites located within SCR1-4 and SCR19-20, while simultaneously binding to surface anionic markers at sites located within SCR6-8 and SCR20. SCR20 of CFH has also been reported to additionally bind to C3d. (Right) CFHR1 can also form a ternary complex by dually engaging C3d/C3b and GAGs. CFHR1 competes with CFH SCR19-20 for binding to the TED, and also sterically inhibits engagement of SCR1-7. CFHR1 is represented in its various characterized physiologic oligomeric states, but additionally forms hetero-dimers with CFHR2 and CFHR5 [22, 23].

Finally, we decided to assess that if in addition to competing with CFH SCR19-20 for C3b TED-binding, CFHR1 can also disrupt the other major interaction that CFH undertakes with C3b. This interaction describes a large discontinuous interface between SCR1-4 and multiple domains of C3b, with SCR4 in particular making contacts with the C3b TED at a location proximal to the site for CFH SCR19-20/CFHR1-binding [28]. To test whether CFHR1-binding to C3b blocks binding of CFH SCR1-4 to C3b we generated recombinant CFHL-1 protein, which apart from the four C-terminal amino acids, is identical to SCR1-7 of CFH. Using a competition-based assay similar to that described above for CFH we found that our wild-type CFHR1 protein was able to disrupt the engagement of CFHL-1 to C3b in a dose-dependent manner (Fig 6). To subsequently rule out the existence of a second binding site for CFHR1 on C3b we then investigated the ability of CFHR1 to bind to complement component C3c. Complement component C3c is a downstream product of C3b proteolysis (requiring complement factor I and complement receptor type 1 as a cofactor) which exhibits the same core arrangement as C3b, except that the TED and CUB domains are absent (S2 Fig) [44]. However, we were unable to detect any binding using an assay in which different concentrations of C3c were immobilized on a plate prior to incubation with CFHR1 (S2 Fig). In agreement with these data, a separate study has also been unable to detect binding of CFHR1 to C3c using a similar ELISA-based method or by surface plasmon resonance (SPR) [34]. It is notable, however, that parts of the N-terminal and the C-terminal binding sites for CFH on C3b are located in close proximity to each other (S2 Fig). Therefore, when CFHR1 binds to the C3b TED, it is likely that the molecule not only directly blocks the CFH SCR19-20 interaction but is also large enough to disrupt SCR1-4 of CFH or CFHL-1 from binding effectively.

The deregulation of CFHL-1 by CFHR1 may be pertinent to disease states associated with complement dysregulation. In particular, a recent study has indicated that in Bruch’s membrane in the eye, CFHL-1 may act as the predominant regulator of complement activation [45]. As genetic data indicate that a commonly occurring deletion of the genes encoding CFHR1 and CFHR3 associates with protection for age-related macular degeneration, a disease state which is linked with uncontrolled complement activation, competition with CFHL-1 for binding to C3b by CFHR1 (or CFHR3) may be pertinent to the development of complement-driven pathology within the eye [20, 46, 47].

The physiological functions of CFHR1 are still unclear. CFHR1 has previously been posited to act as an alternative pathway regulator at the level of C5, inhibiting C5 convertase activity, and exhibiting binding to C5b and to the C5 activation product, C5b6 [17]. A subsequent study, however, has been unable to observe binding to C5 or any functional data consistent with complement regulation at the level of C5 [23]. In our own preliminary work we have been able to detect weak binding between CFHR1 and both C5b and C5b6 using a plate-based methodology (data not shown). However, two separate studies have posited compelling evidence that CFHR1 (along with CFHR2 and CFHR5) may act as antagonists of CFH/CFHL-1 by competing for binding to C3b [22, 23]. Certainly, CFHR1 and CFH/CFHL-1 are found in comparable levels in circulation, and wild-type CFHR1 and CFH have been reported to bind to surface-bound C3b in a comparable manner, with both proteins exhibiting rapid on- and off-rates [22]. However, while CFH demonstrates significantly reduced binding for iC3b, C3dg and C3d, CFHR1 retains its affinity for these smaller C3 proteolytic fragments [22]. Interestingly, the physiological consequences of the interactions that CFHR1 undertakes with iC3b, C3dg and C3d are as yet unknown.

During this study we have been able to isolate the major binding interaction between CFHR1 and complement components C3b and C3d to a single shared site which is identical to that described by the CFH SCR19-20:C3d complex [6, 9]. We have also been able to confirm previously reported data which indicates that dimerization/oligomerization of CFHR1 (and CFHR2 and CFHR5) is necessary for this molecule to effectively bind to C3b/C3d, and to compete with CFH for binding to C3b. Finally, we have been able to establish that CFHR1 can sterically inhibit the interaction that CFH/CFHL-1 SCR1-4 makes with C3b. Therefore, CFHR1 not only directly competes with the C-terminal region of CFH for binding to the TED, but may also disrupt binding of the region of CFH which houses its complement regulatory activity to the rest of C3b. Similar to CFH SCR19-20, CFHR1 can form a ternary complex with both C3b/C3d and heparin (Fig 6 & [17]). In conjunction with the available structures of the CFHR1 SCR1-2 dimer, and the C-terminal three SCR modules of CFH, which are closely homologous to CFHR1 SCR3-5, and which adopts an overall flexible ‘J’-shape, these data have allowed us to consolidate one possible model of CFHR1-mediated deregulation of CFH/CFHL-1 on an activating surface in which CFHR1 directly competes with or blocks both CFH-binding sites on C3b, while simultaneously engaging self-surface anionic markers (Fig 6) [17, 23, 48].

## Supporting Information

S1 Fig Protein interaction sites on CFH SCR19-20 and on C3d. **(A)** Surface representations of CFH SCR19-20 in complex with C3d (corresponding to the C3b TED) and a separate C3d interaction. This complex is shown in a similar orientation to that shown in Fig 2. CFH SCR19-20 is represented in magenta, and the C3d molecule most likely to approximate to that of the C3b TED is shown in red. The second interaction with C3d is also shown, with the C3d molecule in this case being indicated in cyan. **(B)** The C3b TED/C3d and separate C3d footprints on CFH SCR19-20 are indicated in red and cyan, respectively. The CFH SCR19-20 molecule has been rotated 90 degrees in the *x*-axis from that shown in **(A)**. Interfacing residues identified in the crystal-derived complexes are indicated. Amino acids highlighted within orange boxes are present within the respective C3b TED/C3d and C3d interfaces and have also previously been targeted for site-directed mutagenesis studies. The influence that mutations targeting these residues have on the ability of CFH SCR19-20 to bind C3d, C3dg and C3b are summarized within S1, S2 and S4 Tables. **(C)** The CFH SCR19-20 footprint on the C3d molecule identified in the crystal-derived complexes that most likely approximates to the C3b TED is indicated in magenta. In this case, the C3d moiety has been rotated by 270 degrees in the *x*-axis from that shown in **(A)**. Again, interfacing residues are indicated and those which have additionally been targeted by prior mutagenesis studies are highlighted in orange. The effects that mutations targeting these residues have on the abilities of recombinant forms of C3d and C3dg to engage CFH SCR19-20 are summarized in S1 and S3 Tables. **(D)** The CFH SCR20 footprint on the separate C3d moiety is shown. As for **(C)**, the C3d molecule has been rotated by 270 degrees in the *x*-axis from that shown in **(A)**. Residues are labeled according to the same nomenclature employed above for **(B)** and **(C)**. The effects that mutations targeting this binding site have on CFH SCR19-20 binding to C3d/C3dg are summarized in S1 and S3 Tables. **(E)** The physiologic complement receptor type 2 (CR2) SCR1-2 binding site on C3d is indicated. In this case the C3d molecule is shown in an identical orientation to that shown in **(D)**. The CR2 SCR1-2 binding site directly overlaps with that described for CFH SCR20.
(TIF)

S2 Fig CFHR1 interacts only with the C3b TED/C3d. **(A)** Shown is a ribbon representation of the three-dimensional structures of complement components C3c, C3b and C3d (C3c, PDB ID: 2A74; C3b, PDB ID: 2I07; C3d, PDB ID: 1C3d) [29, 44, 49]. C3c is shown in yellow, while the C3b and C3d are shown in red. The core arrangement of C3b is conserved in the smaller C3c fragment (macroglobulin domains 1–8 (MG1-8), a linker domain (LNK) and the carboxyl terminal C345C domain. However, the TED and the CUB domains are absent in C3c. **(B)** When serum-derived C3c at concentrations of 18 pmols, 36 pmols and 72 pmols were immobilized on a plate no binding to CFHR1 could be detected above that observed for BSA (over a concentration range of 0.008 μM -1.04 μM). **(C)** Superposition of the C3b:CFH SCR1-4 and the C3d:CFH SCR19-20 complexes (PDB ID: 2WII and PDB ID: 3OXU, respectively) demonstrating that the C-terminal CFH interaction site on C3b is in close proximity to the N-terminal CFH interaction site [9, 28].
(TIF)

S1 Table Reported SPR steady state binding affinities of wild-type and mutant forms of CFH SCR19-20 for recombinant and plasma-derived forms of wild-type and mutant forms of C3d amine coupled to a CM5 (carboxymethylated dextran surface) sensor chip targeting the separate C3b TED/C3d and C3d interaction sites highlighted in Fig 3 and in S1 Fig. Values taken from Morgan, Schmidt *et al*., (2011) *Nature structural & molecular biology* 18, 463-U101 [9]. * denotes an extrapolated *K*_D_ value which lies outside the concentration range utilized. A dash means the indicated mutant proteins were not assayed. ^†^ denotes *K*_D_ values were measured on a separate chip to the other C3d mutations. Wild-type C3d and the C3d mutants, E1110A, E1110A/D1115A, D1115A, and E1153A described above are identical to those employed in the current study.
(DOCX)

S2 Table Reported SPR binding affinities of recombinant and plasma-derived forms of C3d and C3b, respectively, for immobilized wild-type and mutant forms of CFH SCR19-20 which have been amine coupled to a CM5 sensor chip. Values were taken from Kajander *et al*., Proceedings of the National Academy of Sciences of the United States of America (2011), 108, 2897–2902) [6].
(DOCX)

S3 Table Reported influence of C3dg mutations targeting separate C3b TED/C3d and C3d binding sites on CFH SCR19-20 compared to that of wild-type C3dg. Data were measured by SPR as described above for S2 Table, and also by a separate assay in which binding of ^125^I-labeled CFH SCR19–20 to plate-coated wild-type and mutant forms of C3dg was assessed. Values were taken from Kajander *et al*., Proceedings of the National Academy of Sciences of the United States of America (2011), 108, 2897–2902) [6].
(DOCX)

S4 Table Reported Influence of mutant forms CFH SCR19–20 targeting the separate C3b TED/C3d and C3d binding sites binding to C3b compared to that of wild-type CFH SCR19-20. C3b was conjugated by amine coupling to a CM5 sensor chip, or biotinylated and then immobilized on streptavidin-coated sensor chip. Also shown are the relative abilities of unlabeled mutant CFH SCR19-20 proteins, compared to that of unlabeled wild-type CFH SCR19-20, to compete with I^125^-labeled wild-type CFH SCR19-20 for binding to C3b-coated Zymosan particles. Taken from Ferreira *et al*., Journal of immunology (2009) 182, 7009–7018) [31]. In a separate study, a W1183L mutant form of CFH SCR19-20 also exhibited reduced binding to C3b immobilized on a CM5 sensor chip (Jokiranta *et al*., EMBO journal (2006) 25, 1784–1794) [32].
(DOCX)
