# Supplementary figures and images for: Mapping the Complement Factor H-Related Protein 1 (CFHR1):C3b/C3d Interactions
Source: PLoS One. 2016 Nov 4;11(11):e0166200. doi: 10.1371/journal.pone.0166200 (PMC5096715; doi:10.1371/journal.pone.0166200)

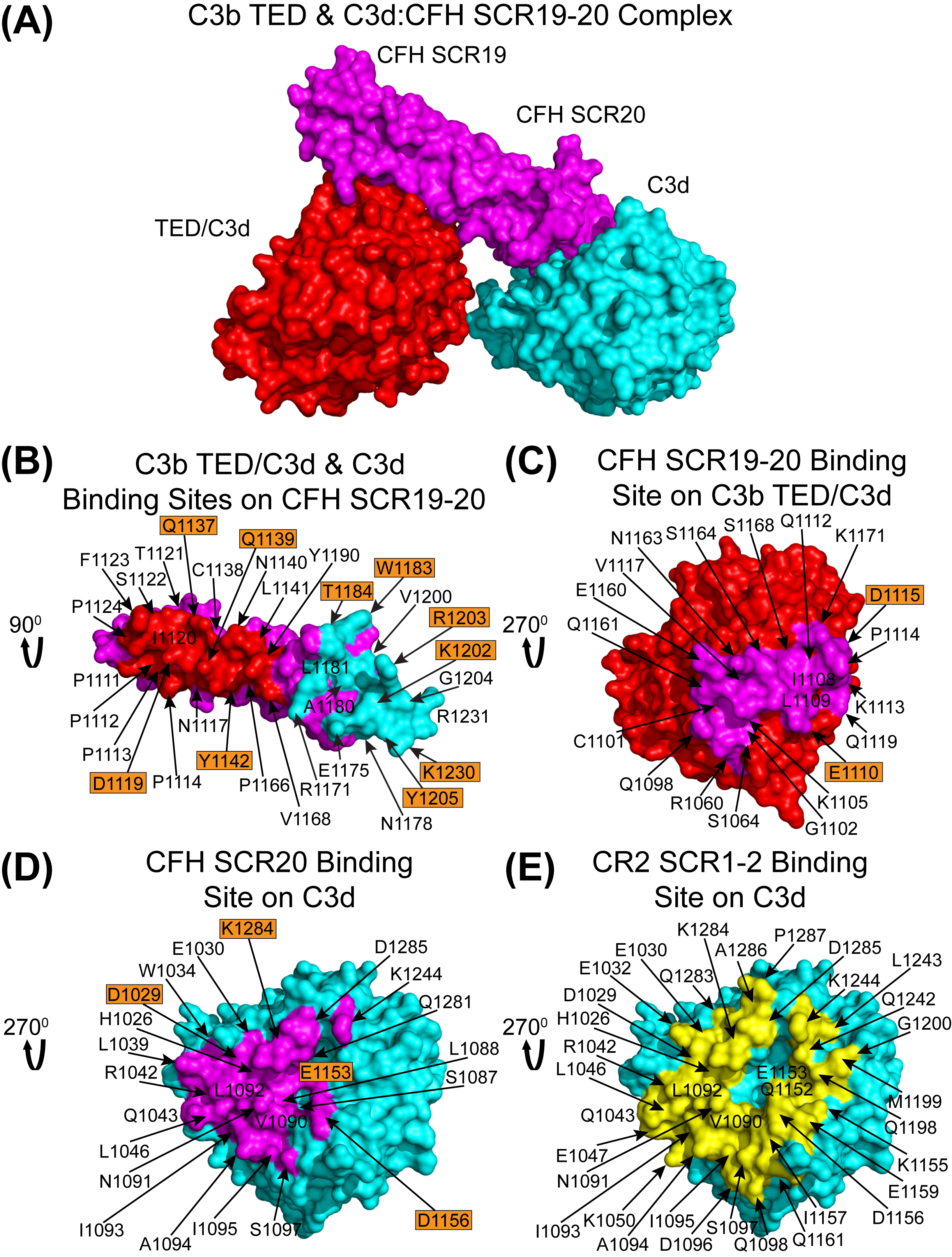

Supplement: S1 Fig — (A) Surface representations of CFH SCR19-20 in complex with C3d (corresponding to the C3b TED) and a separate C3d interaction. This complex is shown in a similar orientation to that shown in Fig 2. CFH SCR19-20 is represented in magenta, and the C3d molecule most likely to approximate to that of the C3b TED is shown in red. The second interaction with C3d is also shown, with the C3d molecule in this case being indicated in cyan. (B) The C3b TED/C3d and separate C3d footprints on CFH SCR19-20 are indicated in red and cyan, respectively. The CFH SCR19-20 molecule has been rotated 90 degrees in the x-axis from that shown in (A). Interfacing residues identified in the crystal-derived complexes are indicated. Amino acids highlighted within orange boxes are present within the respective C3b TED/C3d and C3d interfaces and have also previously been targeted for site-directed mutagenesis studies. The influence that mutations targeting these residues have on the ability of CFH SCR19-20 to bind C3d, C3dg and C3b are summarized within S1, S2 and S4 Tables. (C) The CFH SCR19-20 footprint on the C3d molecule identified in the crystal-derived complexes that most likely approximates to the C3b TED is indicated in magenta. In this case, the C3d moiety has been rotated by 270 degrees in the x-axis from that shown in (A). Again, interfacing residues are indicated and those which have additionally been targeted by prior mutagenesis studies are highlighted in orange. The effects that mutations targeting these residues have on the abilities of recombinant forms of C3d and C3dg to engage CFH SCR19-20 are summarized in S1 and S3 Tables. (D) The CFH SCR20 footprint on the separate C3d moiety is shown. As for (C), the C3d molecule has been rotated by 270 degrees in the x-axis from that shown in (A). Residues are labeled according to the same nomenclature employed above for (B) and (C). The effects that mutations targeting this binding site have on CFH SCR19-20 binding to C3d/C3dg are summari [file pone.0166200.s001.tif]

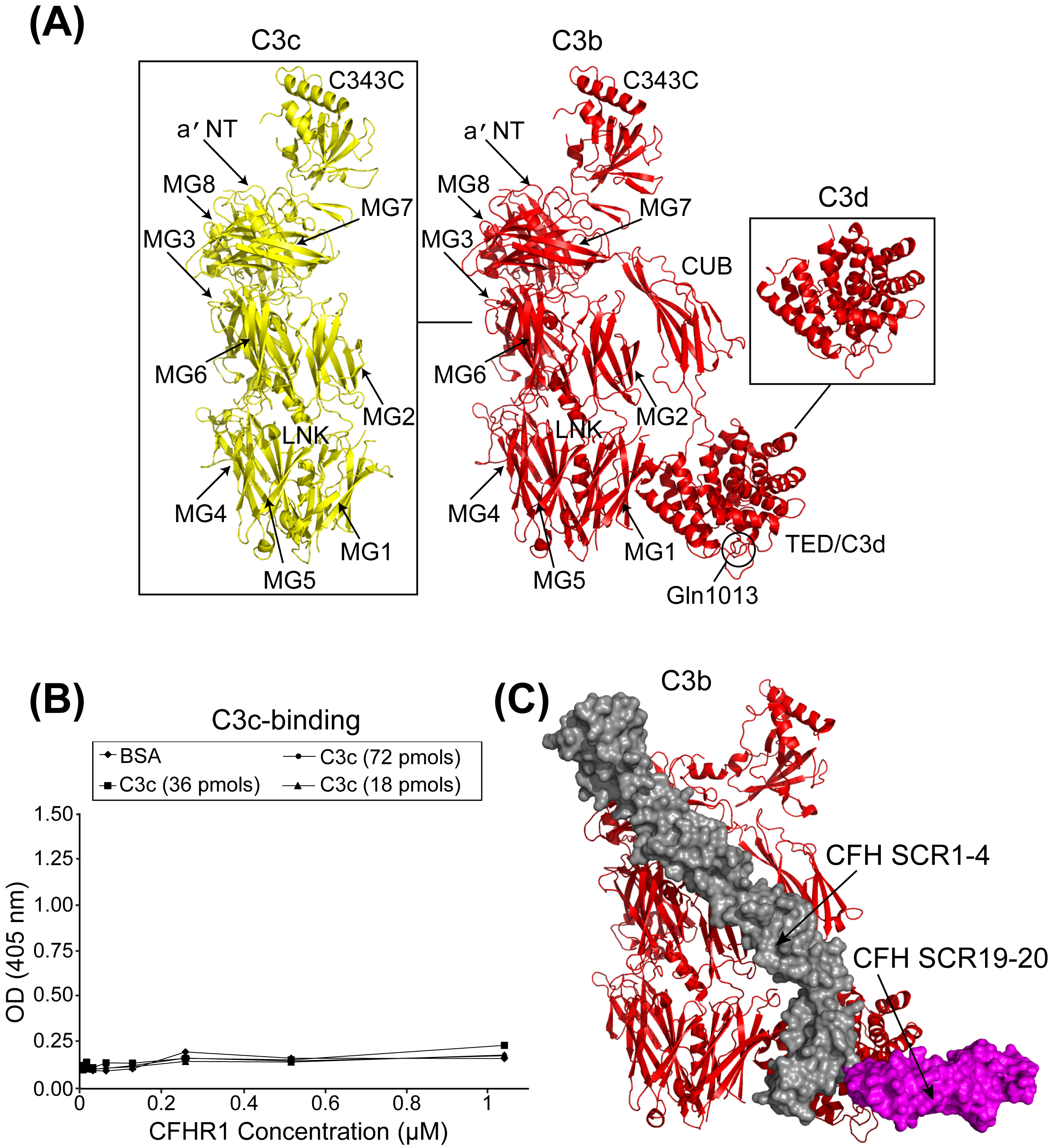

Supplement: S2 Fig — (A) Shown is a ribbon representation of the three-dimensional structures of complement components C3c, C3b and C3d (C3c, PDB ID: 2A74; C3b, PDB ID: 2I07; C3d, PDB ID: 1C3d) [29, 44, 49]. C3c is shown in yellow, while the C3b and C3d are shown in red. The core arrangement of C3b is conserved in the smaller C3c fragment (macroglobulin domains 1–8 (MG1-8), a linker domain (LNK) and the carboxyl terminal C345C domain. However, the TED and the CUB domains are absent in C3c. (B) When serum-derived C3c at concentrations of 18 pmols, 36 pmols and 72 pmols were immobilized on a plate no binding to CFHR1 could be detected above that observed for BSA (over a concentration range of 0.008 μM -1.04 μM). (C) Superposition of the C3b:CFH SCR1-4 and the C3d:CFH SCR19-20 complexes (PDB ID: 2WII and PDB ID: 3OXU, respectively) demonstrating that the C-terminal CFH interaction site on C3b is in close proximity to the N-terminal CFH interaction site [9, 28]. (TIF) [file pone.0166200.s002.tif]
