# Supplementary material for: Mapping the Complement Factor H-Related Protein 1 (CFHR1):C3b/C3d Interactions
Source: PLoS One. 2016 Nov 4;11(11):e0166200. doi: 10.1371/journal.pone.0166200 (PMC5096715; doi:10.1371/journal.pone.0166200)
Supplement: S1 Table — Values taken from Morgan, Schmidt et al., (2011) Nature structural & molecular biology 18, 463-U101 [9]. * denotes an extrapolated KD value which lies outside the concentration range utilized. A dash means the indicated mutant proteins were not assayed. † denotes KD values were measured on a separate chip to the other C3d mutations. Wild-type C3d and the C3d mutants, E1110A, E1110A/D1115A, D1115A, and E1153A described above are identical to those employed in the current study. (DOCX) [file pone.0166200.s003.docx]

| **Reported SPR binding affinities (µM) between CFH SCR19-20 and C3d and C3dg.** (Adapted from Morgan, Schmidt *et al*., *Nature structural & molecular biology.* (2011) 18, 463-U101). | | | | | | | | |
| --- | --- | --- | --- | --- | --- | --- | --- | --- |
|  | | | | **Binding Interface Targeted** | | | | |
|  | | | | **C3b TED/C3d binding Site** | | | **C3d binding site** | |
| **Binding interface targeted** | **CFH Construct** | **Recombinant C3d** | **Plasma-derived C3d** | **C3d E1110A** | **C3d E1110A/D1115A** | **C3d D1115A** | **C3d E1153A** | **C3d I1157A** |
|  | **CFH 19-20** | 6.2 ± 0.4 | 8.2 ± 0.6 | *34 ± 1.0 | *29.8 X 10^3^ ±  1.6 X 10^3†^ | *46.0 ± 5.0 | 15.0 ±2.0 | 14.0 ± 1.4^†^ |
| **C3b TED/C3d binding site** | **CFH SCR19-20 D1119G** | No Binding | No Binding | - | - | - | - | - |
| **C3d binding site** | **CFH SCR19-20 W1183L** | 6.6 ± 0.2 | 8.2 ± 0.3 | - | - | - | - | - |
|  | **CFH SCR19-20 W1183R** | 2.9 ± 0.2 | 4.9 ± 0.4 | - | - | - | - | - |
|  | **CFH SCR19-20 T1184R** | 4.2 ± 0.1 | 6.8 ± 0.2 | - | - | - | - | - |
|  | **CFH SCR19-20 R1203S** | 11.4 ± 0.4 | 13.7 ± 0.3 | - | - | - | - | - |

**S1 Table.** Reported SPR steady state binding affinities of wild-type and mutant forms of CFH SCR19-20 for recombinant and plasma-derived forms of wild-type and mutant forms of C3d amine coupled to a CM5 (carboxymethylated dextran surface) sensor chip targeting the separate C3b TED/C3d and C3d interaction sites highlighted in Fig 3 and in S1 Fig. Values taken from Morgan, Schmidt *et al*., (2011) *Nature structural & molecular biology* 18, 463-U101 [[9](#_ENREF_9)]. * denotes an extrapolated *K*_D_ value which lies outside the concentration range utilized. A dash means the indicated mutant proteins were not assayed. ^†^ denotes *K*_D_ values were measured on a separate chip to the other C3d mutations. Wild-type C3d and the C3d mutants, E1110A, E1110A/D1115A, D1115A, and E1153A described above are identical to those employed in the current study.
