# Supplementary material for: Mapping the Complement Factor H-Related Protein 1 (CFHR1):C3b/C3d Interactions
Source: PLoS One. 2016 Nov 4;11(11):e0166200. doi: 10.1371/journal.pone.0166200 (PMC5096715; doi:10.1371/journal.pone.0166200)
Supplement: S2 Table — Values were taken from Kajander et al., Proceedings of the National Academy of Sciences of the United States of America (2011), 108, 2897–2902) [6]. (DOCX) [file pone.0166200.s004.docx]

| **Reported SPR binding affinities (µM) between wild-type and mutant forms of CFH SCR19-20 and C3d or C3b.** (Adapted from Kajander *et al*., Proceedings of the National Academy of Sciences of the United States of America (2011), 108, 2897-2902). | | | |
| --- | --- | --- | --- |
| **Binding interface targeted** | **CFH Construct** | **Recombinant C3d** | **C3b** |
|  | **CFH SCR19-20** | 0.18 | 0.54 |
| **C3b TED/C3d binding site** | **CFH SCR19-20 Q1137A/Q1139A/Y1142A** | 0.41 | 1.84 |
| **C3d binding site** | **CFH SCR19-20 T1184G/K1202A/R1203A/Y1205A** | 1.14 | 1.48 |

**S2 Table.** Reported SPR binding affinities of recombinant and plasma-derived forms of C3d and C3b, respectively, for immobilized wild-type and mutant forms of CFH SCR19-20 which have been amine coupled to a CM5 sensor chip. Values were taken from Kajander *et al.,* Proceedings of the National Academy of Sciences of the United States of America (2011), 108, 2897-2902) [[6](#_ENREF_6)].
