# Supplementary material for: Mapping the Complement Factor H-Related Protein 1 (CFHR1):C3b/C3d Interactions
Source: PLoS One. 2016 Nov 4;11(11):e0166200. doi: 10.1371/journal.pone.0166200 (PMC5096715; doi:10.1371/journal.pone.0166200)
Supplement: S3 Table — Data were measured by SPR as described above for S2 Table, and also by a separate assay in which binding of 125I-labeled CFH SCR19–20 to plate-coated wild-type and mutant forms of C3dg was assessed. Values were taken from Kajander et al., Proceedings of the National Academy of Sciences of the United States of America (2011), 108, 2897–2902) [6]. (DOCX) [file pone.0166200.s005.docx]

| **Reported influence of C3dg mutant binding to CFH19–20, compared to that of wild-type C3dg, as measured by SPR and by plate-based assay.** (Adapted from Kajander et al., Proceedings of the National Academy of Sciences of the United States of America (2011), 108, 2897-2902). | | | |
| --- | --- | --- | --- |
| **Binding interface targeted** | **C3dg construct** | **Increase, no change, or decrease in binding (SPR) ( ↑, -, or ↓ )** | **Increase, no change, or decrease in binding (plate-based) ( ↑, - or ↓ )** |
| **Control** | **C3dg E1030A** | **-** | **-** |
| **C3b TED/C3d binding site** | **C3dg E1110A** | ↓ | ↓ |
|  | **C3dg D1115A** | ↓ | ↓ |
| **C3d binding site** | **C3dg D1029A** | ↓ | ↓ |
|  | **C3dg E1153A** | ↓ | ↓ |
|  | **C3dg D1156A** | ↓ | ↓ |
|  | **C3dg K1284A** | ↓ | ↓ |

**S3 Table.** Reported influence of C3dg mutations targeting separate C3b TED/C3d and C3d binding sites on CFH SCR19-20 compared to that of wild-type C3dg. Data were measured by SPR as described above for S2 Table, and also by a separate assay in which binding of ^125^I-labeled CFH SCR19–20 to plate-coated wild-type and mutant forms of C3dg was assessed. Values were taken from Kajander *et al.,* Proceedings of the National Academy of Sciences of the United States of America (2011), 108, 2897-2902) [[6](#_ENREF_6)].
