# Supplementary material for: Mapping the Complement Factor H-Related Protein 1 (CFHR1):C3b/C3d Interactions
Source: PLoS One. 2016 Nov 4;11(11):e0166200. doi: 10.1371/journal.pone.0166200 (PMC5096715; doi:10.1371/journal.pone.0166200)
Supplement: S4 Table — C3b was conjugated by amine coupling to a CM5 sensor chip, or biotinylated and then immobilized on streptavidin-coated sensor chip. Also shown are the relative abilities of unlabeled mutant CFH SCR19-20 proteins, compared to that of unlabeled wild-type CFH SCR19-20, to compete with I125-labeled wild-type CFH SCR19-20 for binding to C3b-coated Zymosan particles. Taken from Ferreira et al., Journal of immunology (2009) 182, 7009–7018) [31]. In a separate study, a W1183L mutant form of CFH SCR19-20 also exhibited reduced binding to C3b immobilized on a CM5 sensor chip (Jokiranta et al., EMBO journal (2006) 25, 1784–1794) [32]. (DOCX) [file pone.0166200.s006.docx]

| **Reported influence of mutant forms CFH SCR19–20 binding to C3b, compared to that of wild-type CFH SCR19-20, as measured by SPR and also by their ability to compete with wild-type I^125^-labeled CFH SCR19-20 for binding to C3b on Zymosan particles.** (Ferreira *et al*., Journal of immunology (2009) 182, 7009-7018). | | | | |
| --- | --- | --- | --- | --- |
| **Binding interface targeted** | **CFH construct** | **Increase, no change, or decrease in binding (SPR) C3b/CM5 ( ↑, -, or ↓ )** | **Increase, no change, or decrease in binding (SPR) C3b-biotin/SA ( ↑, - or ↓ )** | **Influence on I^125^-labeled CFH SCR19-20 binding to Zymosan particles (compared to that of unlabeled wild-type CFH SCR19-20)** |
| **C3b TED/C3d binding site** | **CFH SCR19-20 D1119G** | ↓ | ↓ | ↑ |
| **C3d binding site** | **CFH SCR19-20 W1183R** | ↑ (not significant) | ↑ | ↓ |
|  | **CFH SCR19-20 T1184R** | ↑ | ↑ | ↓ |
|  | **CFH SCR19-20 R1203S** | ↓ | ↓ | Not carried out |
|  | **CFH SCR19-20 R1203Y** | ↓ | ↓ | Not carried out |

**S4 Table.** Reported Influence of mutant forms CFH SCR19–20 targeting the separate C3b TED/C3d and C3d binding sites binding to C3b compared to that of wild-type CFH SCR19-20. C3b was conjugated by amine coupling to a CM5 sensor chip, or biotinylated and then immobilized on streptavidin-coated sensor chip. Also shown are the relative abilities of unlabeled mutant CFH SCR19-20 proteins, compared to that of unlabeled wild-type CFH SCR19-20, to compete with I^125^-labeled wild-type CFH SCR19-20 for binding to C3b-coated Zymosan particles. Taken from Ferreira *et al*., Journal of immunology (2009) 182, 7009-7018) [[31](#_ENREF_31)]. In a separate study, a W1183L mutant form of CFH SCR19-20 also exhibited reduced binding to C3b immobilized on a CM5 sensor chip (Jokiranta *et al*., EMBO journal (2006) 25, 1784-1794) [[32](#_ENREF_32)].
